# Use of DNA Fingerprinting To Investigate a Multiyear, Multistate Tuberculosis Outbreak

**DOI:** 10.3201/eid0811.020424

**Published:** 2002-11

**Authors:** Peter D. McElroy, Timothy R. Sterling, Cynthia R. Driver, Barry Kreiswirth, Charles L. Woodley, Wendy A. Cronin, Darryl X. Hardge, Kenneth L. Shilkret, Renee Ridzon1

**Affiliations:** * Centers for Disease Control and Prevention, Atlanta, Georgia, USA; † Baltimore City Health Department, Baltimore, Maryland, USA; ‡ New York City Department of Health, New York City, New York, USA; § Public Health Research Institute, New York City, New York, USA; ¶ Maryland State Department of Health and Mental Hygiene, Baltimore, Maryland, USA; # New Jersey Department of Health and Senior Services, Trenton, New Jersey, USA

**Keywords:** tuberculosis, *Mycobacterium tuberculosis*, outbreaks, DNA fingerprinting, transgender

## Abstract

In 1998–1999, the Baltimore TB control program detected a cluster of 21 tuberculosis (TB) cases. Patients reported frequent travel to various East Coast cities. An investigation was conducted to determine whether transmission of the same *Mycobacterium tuberculosis* strain was occurring in these other localities. A collaborative investigation among federal, state, and local TB controllers included TB record reviews, interviews of patients, and restriction fragment length polymorphism (RFLP) analysis of selected *M. tuberculosis* isolates from diagnosed TB patients in several cities in 1996–2001. A national TB genotyping database was searched for RFLP patterns that matched the outbreak pattern. Eighteen additional outbreak-related cases were detected outside of Baltimore—the earliest diagnosed in New Jersey in 1996, and the most recent in New York City in late 2001. The outbreak demonstrates the need for strategies to detect links among patients diagnosed with TB across multiple TB control jurisdictions.

 Tuberculosis (TB) rates have been declining in the United States since 1993; they reached a low of 5.6 cases per 100,000 population in 2001 (1). To continue this downward trend and eventually achieve the national goal of TB elimination (<1 case per 1 million population per year), ongoing transmission of *Mycobacterium tuberculosis* in social networks needs to be detected earlier (2). When transmission patterns include interstate travel, the epidemiologic connections among TB patients from different localities are often unrecognized, thus inhibiting the ability of local TB controllers to identify a widespread outbreak. New methods that overcome this logistical difficulty will facilitate TB control in this era of increased mobility among difficult-to-reach populations.

 Genetic typing methods to differentiate strains of *M. tuberculosis* are useful in identifying disease clusters resulting from recent transmission (3–5). Beginning in August 1999, the Centers for Disease Control and Prevention (CDC) facilitated a cross-jurisdictional investigation of a TB outbreak first recognized in Baltimore, Maryland, and described by Sterling et al. (6). We present results of this extended investigation that uncovered 18 additional outbreak cases after the original 21 were described. Most of these additional patients lived outside the state of Maryland. The outbreak demonstrates the value of a system that allows *M. tuberculosis* strains to be compared across TB control jurisdictions, particularly in situations where unique social and cultural circumstances hinder conventional approaches to contact investigations and control efforts.

## Methods

### Baltimore Outbreak Profile

Between June 1998 and June 1999, TB was diagnosed in 13 young-adult, U.S.-born African-Americans in Baltimore, Maryland; 8 (62%) were HIV positive. Their *M. tuberculosis* isolates shared a common 11-band DNA fingerprint (6). Upon a review of the National Tuberculosis Genotyping and Surveillance Network database (7), two additional Maryland patients were found to have isolates matching this cluster. Baltimore investigators also suspected that two TB patients in New York City were part of this cluster, a fact that was later verified through genotyping of the patients’ isolates. Among these patients with culture-confirmed TB (age range 18–35 years), all but one were associated with the transgender community (6). The transgender persons were members of a “house,” a social organization of young-adult transgender persons. House members do not necessarily reside together but regularly engage in dance and dressing competitions known as “balls.” A network of several dozen houses exists on the U.S. East Coast (McElroy, unpub. data). Owing to house members’ reported travel patterns, Baltimore investigators suspected that transmission of this same 11-band strain was occurring in other areas, particularly New York City, New Jersey, Atlanta, Boston, Philadelphia, San Francisco, and Washington, D.C. In August 1999, CDC epidemiologists initiated an interstate investigation of this outbreak.

### Genetic Typing

Restriction fragment length polymorphism (RFLP) analysis (DNA fingerprinting) of *M. tuberculosis* isolates was performed according to standard methods (8). RFLP patterns were considered to match if the patterns were identical or differed by the addition or subtraction of a single band.

### Search of National Tuberculosis Genotyping Network Database

An image of the 11-band Baltimore outbreak strain was compared to RFLP images in the TB genotyping network database at CDC. This database contains >6,000 unique fingerprint images collected in the period January 1996–December 2000 from Arkansas, Maryland, Massachusetts, Michigan, New Jersey, five counties in Texas, and the San Francisco Bay area. Images were analyzed with BioImage Whole Band Analyzer software, version 3.4 (Genomic Solutions, Inc, Ann Arbor, MI). Pairs of patterns were compared for matching bands by using a deviation of ±2.5% for molecular weight of each band. Patterns were clustered by using the unweighted pair group method with arithmetic averages (UPGMA/Average) linkage. All matches were verified by visual comparison.

### New York City Investigation

Before 2001, New York City did not routinely perform RFLP analysis. RFLP was retrospectively performed on isolates obtained from 1998 to 2000 from TB patients with demographic characteristics similar to those of the Baltimore outbreak patients (9). After New York City patients associated with the outbreak were identified, their addresses were cross-matched with the entire New York City TB registry. RFLP analysis was performed on isolates from any patient with a street address matching an outbreak-related case. Beginning in January 2001, RFLP analysis was routinely performed on isolates from all TB patients diagnosed in New York City.

### Other City Investigations

In Atlanta and Philadelphia, TB charts of patients fitting the outbreak profile were reviewed, and RFLP analyses were performed on isolates from patients strongly suspected of being part of the outbreak. All isolates from Boston and San Francisco are included in the National Tuberculosis Genotyping and Surveillance Network database, which was searched for matches. San Francisco, although part of the genotyping network, also had a database of additional images from cases preceding 1996. Health authorities in Washington, D.C., did not participate in this investigation.

### Epidemiologic Investigations

A member of the outbreak investigation team contacted patients with isolates found to have an RFLP pattern matching the outbreak strain. Patients or next-of-kin (for deceased persons) were interviewed, either by telephone or in person. A questionnaire was used to assess regular travel destinations, ball attendance, cross-dressing behaviors, and membership in a house.

## Results

As of December 31, 2001, this TB outbreak included 39 patients (36 with culture-confirmed disease) from New Jersey, Maryland, New York City, and Baltimore (Figure). RFLP analysis of the 36 *M. tuberculosis* isolates indicated a matching 11-band pattern in 34 isolates; 2 isolates shared the same 11-band pattern plus 1 additional band. All isolates were susceptible to first-line anti-TB medications. Clinical and demographic characteristics are presented in Tables 1 and 2, respectively.

**Figure:**
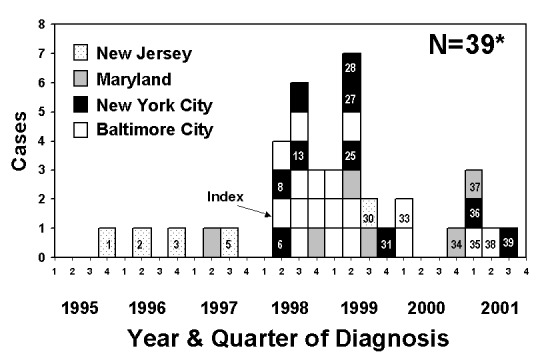
Epidemic curve representing 38 tuberculosis patients associated with an outbreak involving the cities of Baltimore and New York and the states of Maryland and New Jersey, 1995−2001. *Numbered boxes represent additional patients detected after the investigation was extended beyond Baltimore (August 1999). Unnumbered patients (and patient 28) were previously described by Sterling et al. (6).

**Table 1:** Clinical characteristics of tuberculosis outbreak patients, New Jersey, New York City, Baltimore, and Maryland, 1995–2001

Characteristic	New Jersey n=5	New York City n=10	Baltimore n=18	Maryland n=6	Total n=39 (%)
Culture-positive	4	10	17	5	36 (92)
Sputum smear–positive	1	4	9	3	17 (44)
Disease site					
Pulmonary only	2	5	11	4	22 (56)
Exrapulmonary only	0	2	4	2	8 (21)
Pulmonary-extrapulmonary	3	3	3	0	9 (23)
Cavitary disease	0	0	2	1	3 (8)
HIV status					
Positive	2	7	11	1	21 (54)
Unknown	1	0	1	1	3 (8)
Deceased^a^	2	2	1	1	6 (15)

^a^Four of six patients died within 3 months of their TB diagnosis.

**Table 2:** Demographic and social characteristics of tuberculosis outbreak patients, New Jersey, New York City, Baltimore, and Maryland, 1995–2001

Characteristic	New Jersey^a^	New York City	Baltimore	Maryland	Total	
	n=5	n=10	n=18	n=6	n=39 (% of total)	
Median age, yrs (range)	20 (6–33)	30 (1–40)	24 (19–43)	33 (21–46)	26	(1–46)
						
African-American	4	9	18	5	36 (92)	
						
Born as male	2	10	14	4	30 (77)	
						
House member	1	7	11	0	19 (49)	
						
Pediatric patient	2	1	0	0	3 (8)	
						
Foreign born	1	0	1	0	2 (5)	

^a^Probable nosocomial exposure for one of these two patients occurred at a New Jersey hospital before the patient’s relocation to Maryland.

### New Jersey

The initial search of the 6,000 RFLP images in the network database in August 1999 yielded two New Jersey isolates that matched the outbreak strain. The earliest matching RFLP pattern came from an isolate cultured in November 1996 from a 17-year-old woman (patient 3), the sister of a culture-negative 7-year-old girl (patient 2) with clinical TB that had been diagnosed earlier that year (Figure). The second match was an isolate from a 33-year-old woman whose TB was diagnosed in 1997 (patient 5). These three patients were contacts from either the household (patients 2 and 3) or workplace (patient 5) of a 24-year-old man (patient 1) in whom pulmonary TB was diagnosed in 1995, one year preceding initiation of the genotyping network in New Jersey. Thus, although no isolate was available from case-patient 1 at the time of the 1999–2000 outbreak investigation, he was epidemiologically linked to this outbreak. This patient reported a history of frequent travel to New York, Baltimore, Philadelphia, and Atlanta to attend and compete in balls but denied ever being a member of a house and had no identified link to any outbreak cases. The most recent New Jersey patient associated with this outbreak was a transsexual who had undergone male-to-female postoperative surgery (patient 30); her case of TB had been diagnosed in September 1999, and she died in early 2000. As of this writing (mid-2002), no other New Jersey isolates match the outbreak strain.

### New York City

RFLP analyses performed on 235 isolates from selected patients diagnosed in 1998–2000 identified seven additional patients (nos. 6, 8, 13, 25, 27, 31, and 36) not previously recognized as being associated with each other or any outbreak case-patients in Baltimore. Interviews indicated that all patients but one (patient 31) were part of the same transgender network described in the Baltimore outbreak. TB was diagnosed in patient 6 in the same month as the case in the Baltimore index patient. The address cross-match identified patient 31, a 1-year old boy who resided in an apartment across the hallway from patient 28. The child’s isolate had an RFLP pattern matching the outbreak strain. Neither patient’s list of identified contacts included the other patient’s name.

Since January 2001, two additional outbreak cases have been identified from New York City after implementation of universal RFLP analysis for isolates obtained from all TB patients in that city. Patient 36 is the only Latino thus far associated with the outbreak. The latest known patient associated with this outbreak (patient 39) was an HIV-coinfected transsexual who had not undergone sex change surgery; plural TB was diagnosed in September 2001.

### Maryland

Two additional cases were diagnosed in Maryland after the original report, both in persons from counties bordering Baltimore City. A 42-year-old man with meningeal TB (patient 34) died before he could be interviewed regarding his association with other persons in the outbreak, and his family has not cooperated with this investigation. A 46-year-old woman (patient 37) relocated from New Jersey to Maryland just before her TB diagnosis. The RFLP pattern of her isolate matched the outbreak strain, and subsequent interviews (corroborated by employment records) revealed that in 1996 she worked as a custodian at the same county hospital in New Jersey where patient 1 was hospitalized for TB, on the same floor.

### Baltimore

To date, TB was been diagnosed in three additional patients with isolates matching the outbreak strain in Baltimore since the first report (6). Both male patients (patients 33 and 38) were associated with the transgender network. One female patient (patient 35) had contact with a son of the nightclub owner whose club was frequented by most outbreak patients.

### Other Sites

RFLP analysis performed on available isolates from patients fitting the outbreak profile in Atlanta (n=26) and Philadelphia (n=3) found that none matched the outbreak strain. RFLP patterns of four San Francisco isolates from the pre-1996 database closely resembled the outbreak strain. These isolates were retyped in 2001 but did not match the outbreak strain. No isolates from Massachusetts or other TB genotyping network sites matched the outbreak strain.

## Discussion

 Ongoing transmission of *M. tuberculosis* in this outbreak occurred in multiple jurisdictions for at least 4 years (1996–1999). Early recognition of this outbreak by the City of Baltimore TB Control Program staff and their suspicion of a larger outbreak network beyond that city were critical to the initiation of the multistate investigation. The transient nature of the outbreak’s social network within Baltimore and the members’ propensity for frequent travel and shared accommodations in other cities created an opportunity for extensive transmission of *M. tuberculosis*. Subsequently, the high prevalence of HIV coinfection within this population led to an increased likelihood of progression from latent TB infection to TB disease (10). Through the detection of isolates with matching RFLP patterns, followed by interviews with patients regarding their travel to and participation in transgender social events, TB controllers were able to plan and implement specific interventions for this population. Outbreak-associated cases continue to be detected. Continued transmission of this strain is possible, given that pleural disease was diagnosed in a 29-year-old HIV-coinfected patient in September 2001.

DNA fingerprinting of *M. tuberculosis* isolates has proven to be an extremely useful tool for tracking transmission of various genotypes throughout communities. This technology has a demonstrated role in investigations of transmission within facilities such as prisons (11), hospitals (12), other localized settings such as homeless shelters (13), or in individual states (14). However, few studies have used RFLP to uncover outbreak networks across geographic areas this large. The predictive value of RFLP patterns to document recent *M. tuberculosis* transmission and the relatedness of isolates is often considered stronger in outbreak settings in which links among patients are known (14). This investigation demonstrated that, after a general demographic profile was established among a group of TB patients (e.g., 18- to 35-year-old, HIV-positive, African-American men) from one locality, typing of isolates from other localities allowed investigators to successfully uncover additional matching isolates from patients with characteristics similar to those of the Baltimore cluster. This outbreak demonstrated the value of periodically comparing RFLP fingerprint patterns beyond local jurisdictions to establish whether transmission of a particular strain extends to a broader geographic area. During the second quarter of 1998, four cases with isolates retrospectively matching the outbreak strain were diagnosed in Baltimore and New York City. By early 1998, the national TB genotyping network database already included at least three isolates from New Jersey and Maryland that matched the outbreak strain. Earlier recognition of the common sociodemographic links among the initial case-patients in multiple localities might have averted the subsequent cases diagnosed between 1999 and the present.

 Casual transmission of *M. tuberculosis* is defined as transmission from an infectious TB patient to persons who are not household, work, school, or close contacts (15). This outbreak included three patients with isolates matching the RFLP pattern of the outbreak strain but with no identified epidemiologic link to other cases, despite extensive investigation. One should be cautious about viewing cases with no clear epidemiologic link as evidence of casual transmission. These patients’ denial of any association with the outbreak network may be a consequence of the secretive nature of some transgender persons, who are often ostracized by society. Some of the male outbreak patients appeared as women only on occasion, and otherwise assimilated into their jobs or schools as men. Many of the transgender patients also engaged in commercial sex work, another potential source of exposure for patients with no identifiable ties to other outbreak patients.

 The detection of matching RFLP patterns alone was insufficient to allow investigators to fully characterize the social network in which transmission was occurring. As RFLP analysis of *M. tuberculosis* isolates is performed on a routine basis in large urban areas (e.g., New York City), case-clusters will continue to emerge. As this happens, TB controllers will be expected to direct interventions toward these apparent clusters. Our investigation confirmed that simply detecting a disease cluster, without describing and understanding the social milieu supporting transmission, can lead to incomplete and inefficient TB control. Cutting-edge molecular tools can be enhanced by equally novel epidemiologic approaches. The role of alternative epidemiologic methods such as network analysis, used in tandem with DNA fingerprint analysis, warrants further investigation (16,17).
